# Inflamm-Aging and Arachadonic Acid Metabolite Differences with Stage of Tendon Disease

**DOI:** 10.1371/journal.pone.0048978

**Published:** 2012-11-14

**Authors:** Stephanie Georgina Dakin, Jayesh Dudhia, Natalie Jayne Werling, Dirk Werling, Dilkush Robert Ephrem Abayasekara, Roger Kenneth Whealands Smith

**Affiliations:** 1 Department of Veterinary Clinical Sciences, Royal Veterinary College, University of London, Hatfield, United Kingdom; 2 Department of Biotherapeutics, National Institute for Biological Standards and Control, South Mimms, United Kingdom; 3 Department of Pathology and Infectious Diseases, Royal Veterinary College, University of London, Hatfield, United Kingdom; 4 Department of Veterinary Basic Sciences, Royal Veterinary College, University of London, London, United Kingdom; National Institute of Health (NIH), United States of America

## Abstract

The contribution of inflammation to the pathogenesis of tendinopathy and high prevalence of re-injury is not well established, although recent evidence suggests involvement of prostaglandins. We investigated the roles of prostaglandins and inflammation-resolving mediators in naturally occurring equine tendon injury with disease stage and age. Levels of prostaglandins E_2_ (PGE_2_), F_2α_ (PGF_2α_), lipoxin A_4_ (LXA_4_) and its receptor FPR2/ALX were analysed in extracts of normal, sub-acute and chronic injured tendons. To assess whether potential changes were associated with altered PGE_2_ metabolism, microsomal prostaglandin E synthase-1 (mPGES-1), prostaglandin dehydrogenase (PGDH), COX-2 and EP_4_ receptor expression were investigated. The ability of tendons to resolve inflammation was determined by assessing FPR2/ALX expression in natural injury and IL-1β stimulated tendon explants.

Alterations in the profile of lipid mediators during sub-acute injury included low PGE_2_ and elevated LXA_4_ levels compared to normal and chronic injuries. In contrast, PGF_2α_ levels remained unchanged and were three-fold lower than PGE_2_. The synthetic capacity of PGE_2_ as measured by the ratio of mPGES-1:PGDH was elevated in sub-acute injury, suggesting aberrations in tendon prostaglandin metabolism, whilst COX-2 and EP_4_ receptor were unchanged. Paradoxically low tendon PGE_2_ levels in early injury may be attributed to increased local clearance via PGDH or the class switching of lipid mediators from the prostaglandin to the lipoxin axis. PGE_2_ is therefore implicated in the development of tendon inflammation and its ensuing resolution. Whilst there was no relationship between age and tendon LXA_4_ levels, there was an age-associated decline in FPR2/ALX receptor expression with concurrent increased PGE_2_ levels in injury. Furthermore, uninjured tendon explants from younger (<10 years) but not older horses (≥10 years) treated with IL-1β responded by increasing FPR2/ALX suggesting aged individuals exhibit a reduced capacity to resolve inflammation via FPR2/ALX, which may present a potential mechanism for development of chronic tendinopathy and re-injury.

## Introduction

Tendinopathy of the human Achilles and the functionally equivalent equine superficial digital flexor tendon (SDFT) are significant causes of morbidity in athletic individuals [1], [2]. Repetitive mechanical loading during exercise is cited as a major causative factor [3], [4] with high risk of re-injury [5] due to the inferior mechanical properties of the poorly organised fibrous tissue following healing [6]. The importance of inflammation in tendinopathy is highly debated with the aetiology often cited as a degenerative mechanism [7], [8]. However, this inference is influenced by analyses of injured human tendons that are often only available for examination at surgery, usually some time after the initial injury, by which time acute phase events are lost and chronic disease is well established.

**Figure 1:**
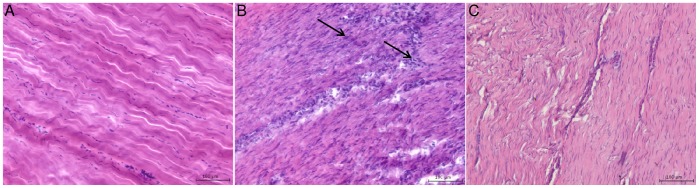
Typical microscopic appearance of normal and injured equine flexor tendons. Longitudinal histology sections stained with Haematoxylin and Eosin showing: (A) normal superficial digital flexor tendon (SDFT) from a 6 year old horse showing regular arrangement of parallel collagen fibrils. Scale bar = 100 µm. (B) Sub-acutely injured SDFT 3 weeks post injury from a 4 year old horse showing marked cellular infiltration (black arrows). Scale bar = 100 µm. (C) Chronic injured SDFT >3 months post injury from a 12 year old horse showing increased cellularity and poor organisation of collagen fibrils compared to (A). Scale bar = 100 µm.

The horse presents an attractive large animal model for the study of the equivalent human injury due to the shared characteristics of aging phenotypes [9], [10] and elastic energy storing function common to the weight-bearing tendons of both species [11], [12]. Equine tendons present a more readily attainable source than the human counterpart, permitting targeted investigation of disease throughout the injury phases as well as normal (uninjured) tendons of a wide age range for comparison. Furthermore, similar to the human injuries, tendon repair processes are frequently clinically classified into three phases in naturally occurring equine injury; the acute phase occurs immediately after the initial trauma lasting only a few days, followed by sub-acute (3–6 weeks) and chronic injury phases (>3 months after injury) [13].The tensile region of the equine SDFT is most susceptible to overstrain injury [14], [15]. Injured tendons are enlarged compared to normal and exhibit a haemorrhagic granular central core during early stage injury. The histological appearance of injured equine SDFTs are shown in Fig. 1, illustrating increased cellularity soon after injury compared to normal tendons. During healing, the damaged tissue is remodelled and a fibrogenic scar repair forms and the highly organised arrangement of collagen fascicles are not restored (Fig. 1c) and [16], predisposing to re-injury due to diminished mechanical strength. The effects of age, exercise and mechanical loading are inextricably linked and are potentially synergistic factors in the development of tendinopathy. The frequency of tendon injury in sprint horses has been shown to increase with age from 6% in 2 year olds to 16% in horses aged 5 years and over [17]. Similarly, an increased incidence of Achilles tendon rupture has also been reported in middle aged athletes or aged non-athletic persons [18], [19]. Hence the effects of ageing and cumulative microdamage can further exacerbate the risk of re-injury in diseased tendons.

The contribution of inflammation to the development of tendinopathy is not fully elucidated and there is a paucity of data reporting inflammatory processes, particularly during the early stages of injury. However, several studies support the involvement of prostaglandins such as prostaglandin E_2_ (PGE_2_) in the development of tendinopathy via inflammatory processes [20]–[22]. Indeed, prostaglandin lipid mediators are synthesised in response to tissue insult or injury and contribute to pain and inflammation in many connective tissues within the body [23]. PGE_2_ levels are reported to increase in the peri-tendinous space of the Achilles of healthy exercising human subjects [24] and in murine patellar and Achilles tendons following treadmill exercise [25], suggesting exercise can also induce tendon inflammation. These observations are supported by *in vitro* experiments whereby tendon fibroblasts in culture release PGE_2_ in response to repetitive cyclic strain [26]–[28]. Furthermore, prostaglandins regulate MMP production, partly via an IL-1β mediated mechanism in catabolism of cartilage, periodontal ligament [29], [30] and tendon [20], [22] contributing to degradation of the extracellular matrix (ECM). However the involvement of other prostaglandins such as those of the D series and their cyclopentanone metabolites to the development of tendinopathy are not known.

The receptors mediating prostaglandin effects are also cited as contributors to the pathogenesis of tendon injuries. A series of four EP receptor subtypes are responsible for the downstream effects of PGE_2_. The EP_4_ receptor is reported to mediate the IL-1β-induced catabolic metabolism via the p38 MAPK pathway in human tendon fibroblasts, implicating its role in the development of tendinopathy [31]. Regulation of mPGES-1 and PGDH enzymes controlling prostaglandin synthesis and the clearance mechanisms associated with degradation have been described for burn related injuries and sepsis in human patients [32]. However, little is currently known about prostaglandin metabolism in flexor tendons that have sustained a natural injury, nor the effect of injury stage and age.

In addition to prostaglandins, other products of the arachadonic acid pathway exert important roles in regulating inflammation. Lipoxin A_4_ (LXA_4_) is a specialised pro-resolving mediator that selectively signals through the FPR2/ALX receptor providing endogenous stop signals for inflammation [33], [34]. The ability to resolve inflammation after injury or sepsis is well documented for other body tissues [33], [35], [36], although knowledge of the anticipated roles of specialised pro-resolving mediators such as lipoxins is limited for tendon injuries. We recently described significantly increased expression of FPR2/ALX in sub-acutely injured equine tendons [16]; however expression appeared to be of insufficient duration and magnitude to suppress inflammation, which may potentiate development of chronic disease and fibrotic repair.

Taking all these observation together, it is likely that additional factors play a role in repair-processes during tendon injury. A reduced ability to respond to inflammation may be a contributing factor influencing the reduced efficacy of tendon repair. Inflamm-aging is a component of immunosenescence which is an age associated decline in immune function, whereby the major cell types of the immune system exhibit age-related changes, resulting in a diminished ability to cope with inflammation [37]. Although tendon pathology and incidence of injury are known to increase in aged individuals [18], [38], the effect of age on the ability to resolve tendon inflammation and the contribution of immunosenescence to the development of disease are not understood. The aims of this study were to assess the temporal and differential alterations in prostaglandin and resolving lipid mediators in normal and naturally injured equine tendons throughout the stages of healing and to determine the effect of age and injury stage on the regulation of prostaglandin metabolism. We hypothesised that the production of PGE_2_ increases with age in injured flexor tendons and that pro-resolving lipid mediators are activated during the early injury phase. We report altered PGE_2_ metabolism and elevated LXA_4_ levels occur during the early stage of tendon disease, and reduced expression of the inflammation resolving receptor FPR2/ALX with increasing age, which has implications for sustaining chronic injury.

## Results

### Class Switching of Lipid Mediators Occurs in Early Stage Tendon Injury

**Figure 2:**
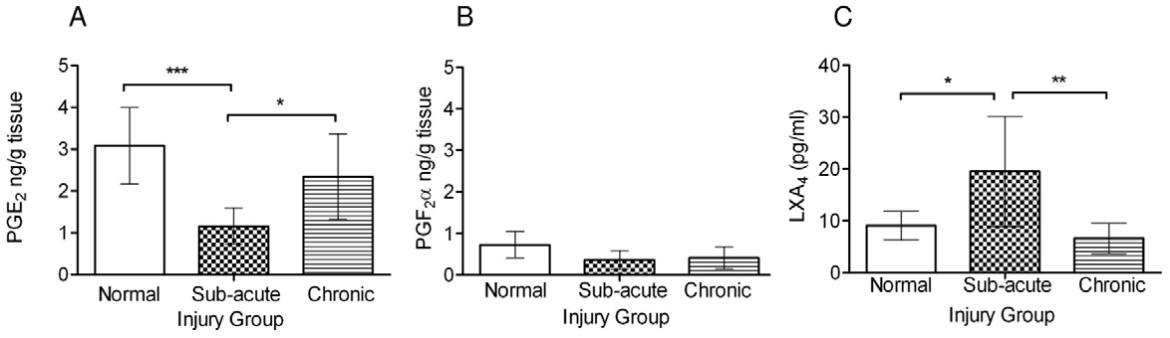
Levels of prostaglandins and lipoxin A_4_ in extracts of normal and injured tendons. (A) Mean PGE_2_ and (B) mean PGF_2α_ levels in normal (n = 19), sub-acute (3–6 weeks post injury, n = 6) and chronic injured (>3 months post injury, n = 9) equine superficial digital flexor tendons. PGE_2_ levels are significantly reduced in sub-acute injury compared to normal and chronic injuries. In contrast PGF_2α_ levels are 3 fold lower than PGE_2_ and do not change with injury. (C) Mean LXA_4_ levels in normal (n = 8), sub-acute (n = 7) and chronic (n = 6) injured tendons, showing significantly increased levels in sub-acute injury compared to normal and chronic injuries. Error bars denote standard deviation. * *P*<0.05, **P<0.01, *** *P*<0.001.

**Figure 3:**
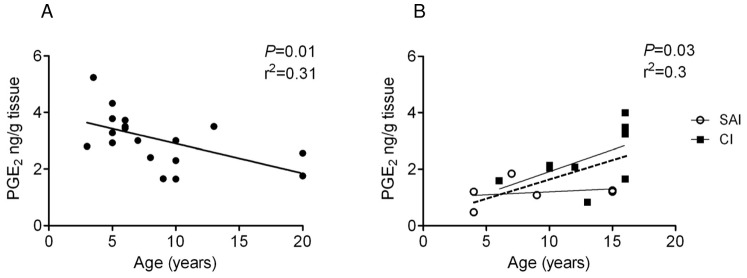
The relationship between horse age and PGE_2_ levels in tendon extracts. (A) Normal flexor tendons (n = 19) showed a significant negative correlation between PGE_2_ levels and increasing age (*P* = 0.01, r^2^ = 0.31). (B) Injured tendons (n = 15) comprised of both sub-acute and chronic injuries showed a significant positive correlation (dotted line) between PGE_2_ levels and increasing age (*P* = 0.03, r^2^ = 0.3) although when separated for injury stage, neither sub-acute nor chronic injuries were significant in isolation (solid lines). SAI =  sub-acute injury (3–6 weeks post injury, n = 6), CI =  chronic injury (>3 months post injury, n = 9).

PGE_2_ concentrations were reduced in extracts prepared from sub-acutely injured tendons compared to normals and chronic injuries (*P*<0.001 and *P*<0.05 respectively) (Fig. 2a). In contrast, PGF_2α_ concentrations were similar in normal and injured tendons and were 3-fold less compared to PGE_2_ (Fig. 2b). Furthermore, increased (∼2-fold) level of LXA_4_ was found in sub-acute injury compared to normal and chronic injured tendons (*P*<0.05; *P*<0.01 respectively) (Fig. 2c), although no correlation was seen between tendon LXA_4_ levels and age within each group. The relationship between PGE_2_ levels with age in normal and injured tendons was also assessed in these samples. In normal tendons, there was a significant negative correlation between PGE_2_ levels and horse age (*P*≤0.01, r^2^ = 0.31) (Fig. 3a). In contrast, with injury there was a significant positive correlation between PGE_2_ levels and increasing horse age (*P*<0.05, r^2^ = 0.3) (Fig. 3b), although when separated for injury stage, neither sub-acute nor chronic injuries were significant in isolation.

### Regulation of Tendon PGE_2_ Metabolism

**Figure 4:**
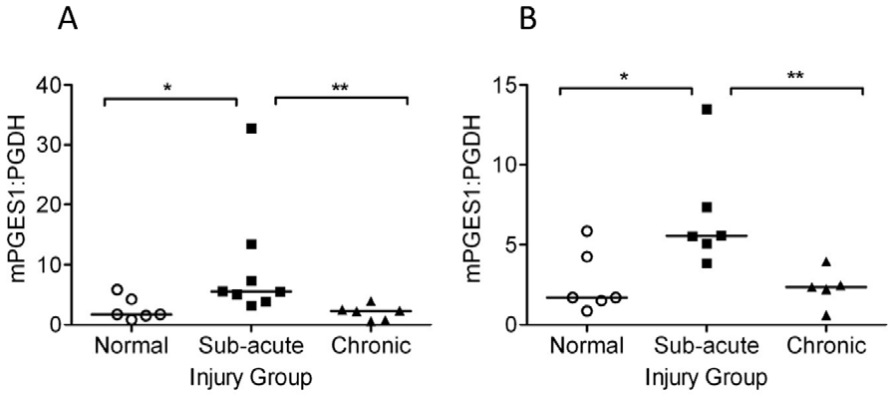
mPGES-1 and PGDH expression in normal and injured tendons. mPGES-1 and PGDH mRNA expression were normalised to GAPDH or 18S ribosomal RNA and are shown expressed as mPGES-1: PDGH ratio in each case. (A) Median values for GAPDH normalized ratio of mPGES-1: PGDH gene expression in normal (n = 6), sub-acute (n = 8) and chronic injured flexor tendons (n = 6). (B) Median values for 18S normalized ratio of mPGES-1: PGDH gene expression in normal (n = 6), sub-acute (n = 6) and chronic injured flexor tendons (n = 5), showing elevated mPGES-1:PGDH expression in sub-acute injury compared to normal and chronic injured tendons.

As PGF_2α_ levels were lower than PGE_2_ and did not differ with injury stage, further analyses were focused towards PGE_2_. To assess whether the measured differences in PGE_2_ were attributable to altered prostaglandin metabolism, we analysed gene expression of the key enzymes responsible for PGE_2_ synthesis (COX-2, mPGES-1) and degradation (PGDH) based on their roles in prostaglandin metabolism. Normalized mPGES-1 and PGDH expression did not change significantly between normal and injured tendons (data not shown). To assess the balance between PGE_2_ synthesis and degradation, we analysed the ratio of the two key enzymes involved in PGE_2_ metabolism mPGES-1 and PGDH at the different stages of injury. This comparison revealed a ∼3-fold increase of mPGES-1:PGDH in sub-acute injury compared to normals (*P*<0.05) and chronic injury (*P*<0.01) (Fig. 4a normalized to GAPDH and 4b normalized to 18S ribosomal RNA). There was no relationship between mPGES-1:PGDH mRNA expression with age and no significant differences were observed in COX-2 or EP_4_ receptor mRNA expression with age or between normal and injured tendons (data not shown).

**Figure 5:**
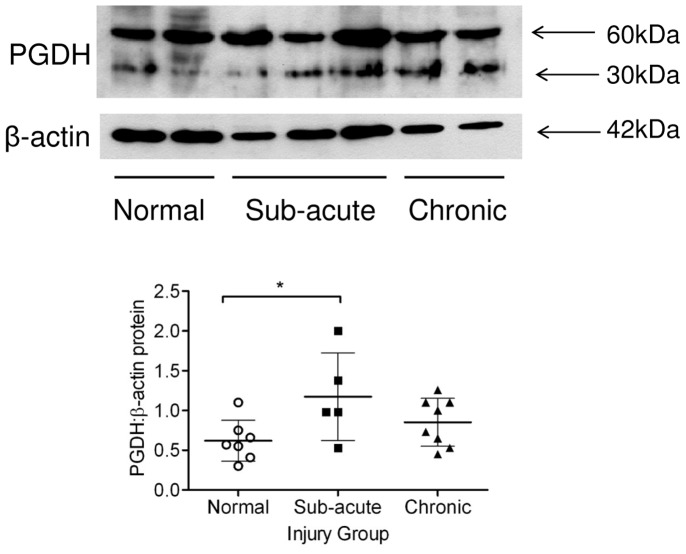
Representative Western blots illustrating expression of PGDH and β-actin in normal, sub-acute and chronic SDFT extracts. Monomeric (30 kDa) and dimeric (60 kDa) bands are shown for PGDH and a 42 kDa band for β-actin. Samples were loaded on a volume basis and the ratio of PGDH normalised to β-actin was calculated for each sample using band densitometric analysis. Graph shows densitometric analysis of western blots for PGDH in protein extracts prepared from normal (n = 7) sub-acute (n = 5) and chronic injured SDFTs (n = 8). The densitometric values were normalized to levels of β-actin expressed in each sample. There was a significant increase in PGDH in sub-acutely injured tendon extracts compared to normals but this was not significantly different in the chronic injury group. * *P*<0.05, **P<0.01. Mean values are shown, error bars denote standard deviation.

PGDH and mPGES-1 proteins were also assessed in extracts of normal, sub-acute and chronic injured SDFTs. A representative Western blot of PGDH protein expression is shown in Fig. 5. Protein bands indicate two forms of PGDH are present in tendons as previously reported in equine preovulatory follicles, showing a minor monomeric form (30 kDa) and a major dimeric form (60 kDa) [39]. Densitometric analysis of Western blots of PGDH normalised to β-actin showed significantly increased PGDH levels in sub-acutely injured tendon extracts compared to normals (*P* = 0.04) (Fig. 5), but this was not significantly different in the chronic injury group. mPGES-1 was detectable at very low level in normal and injured tendon extracts and was not quantifiable (data not shown).

### FPR2/ALX Expression is Upregulated in Natural Tendon Injury and by IL-1β in vitro

**Figure 6:**
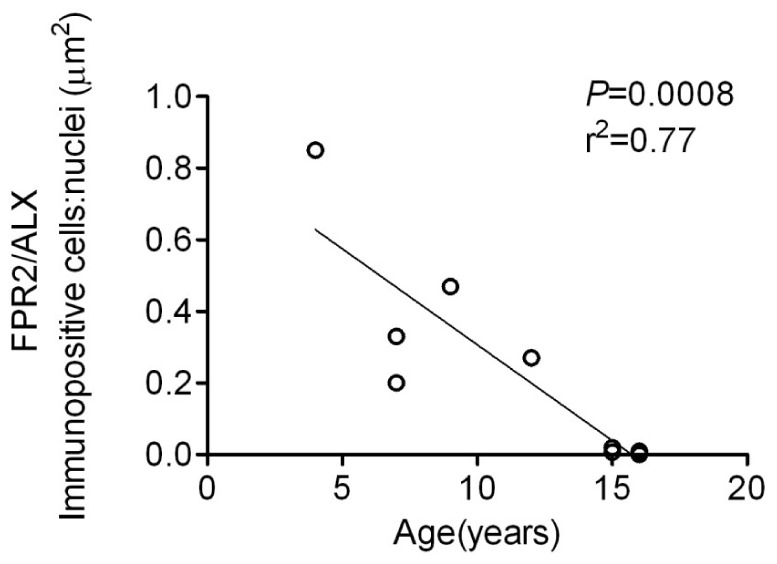
FPR2/ALX protein expression in natural tendon injury. The relationship between FPR2/ALX levels with age is shown in injured flexor tendons (n = 10). Horse age ranged between 4 and 16 years (mean 11±4 years). FPR2/ALX expression was significantly reduced with increasing age (*P* = 0.0008, r^2^ = 0.77). Overlapping points are present for tendons derived from more than one 15 and 16 year old horses.

**Figure 7:**
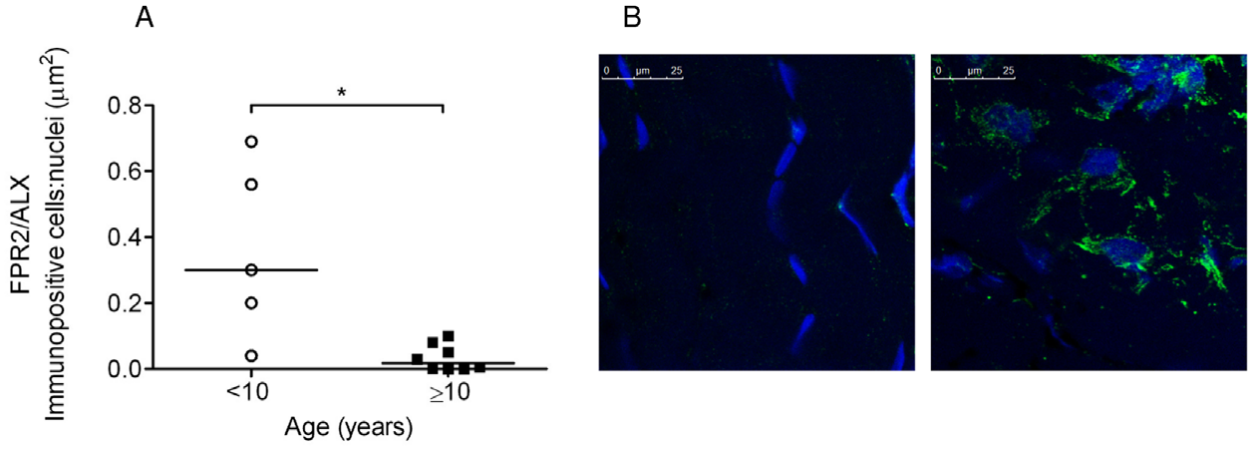
FPR2/ALX protein expression in tendon explants *in vitro*. (A) FPR2/ALX protein expression is shown for IL-1β stimulated macroscopically normal tendon explants derived from horses <10 years of age (n = 5) or ≥ 10 years of age (n = 8). There was significantly greater FPR2/ALX expression by tenocytes in IL-1β stimulated explants from horses less than 10 years of age compared to older horses (*P* = 0.01). Data represent average FPR2/ALX expression whereby 2 replicates were analysed per horse and median values are shown. (B) Panel of representative 2-dimensional confocal images illustrating FPR2/ALX expression in tendon explants from a 4 year old horse showing non-stimulated control (left) compared to stimulation with 5 ngml^−1^ IL-1β (right). FPR2/ALX expression was not detectable in non-stimulated controls. Immunopositive staining is green, with Hoechst nuclear counter stain in blue. Scale bar = 25 µm.

Based on the temporal differences in PGE_2_ levels, we next addressed whether alterations in the pro-resolution mediators FPR2/ALX and LXA_4_ existed with age or disease stage and their response to inflammation. We previously reported FPR2/ALX protein expression was not detectable in uninjured tendons [16]. In the current study we focused on determining FPR2/ALX expression in natural tendon injury and its regulation in cytokine stimulated tendon explants *in vitro*. Linear correlation analysis of tendons from horses with injuries showed a significant negative correlation between FPR2/ALX protein expression and age (*P*<0.001, r^2^ = 0.77) (Fig. 6). Interestingly, its expression was lowest in chronic injuries which mostly occurred in the older animals. To test the hypothesis whether the predominance of chronic injuries with age was related to a diminished ability of tendons to resolve inflammation, FPR2/ALX expression was determined in explant cultures of normal tendons stimulated with 5 ngml^−1^ IL-1β. FPR2/ALX expression could be upregulated by IL-1β in tendons derived from young horses (<10 years old) but its expression was significantly reduced in explants derived from horse’s ≥10 years of age (mean ∼10-fold reduction; *P* = 0.01) (Fig.7a). In contrast, FPR2/ALX expression was not detectable in the corresponding non-stimulated controls (Fig 7b). There was no correlation between media LXA_4_ levels and age from tendon explants stimulated with IL-1β (data not shown).

### LXA_4_ Levels in Media after Combined Stimulation with IL-1β and PGE_2_

**Figure 8:**
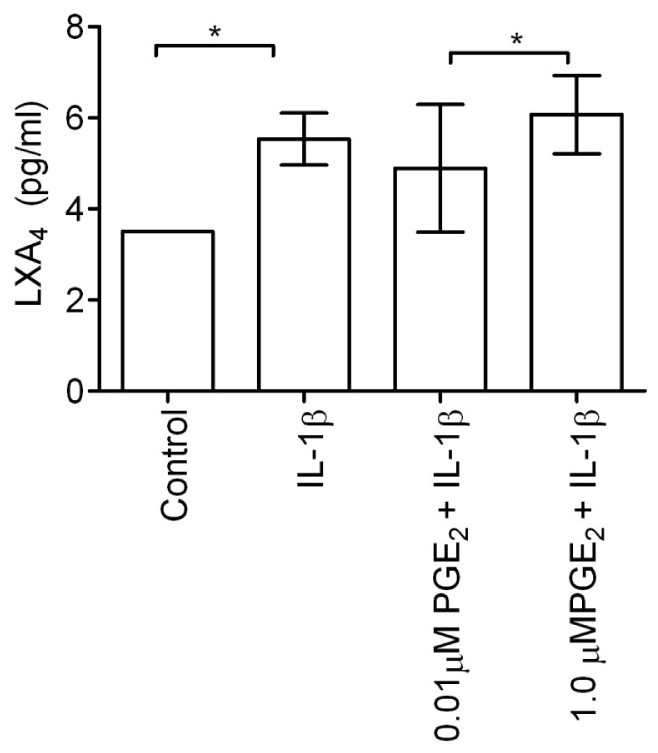
Mean LXA_4_ levels 24 hours after stimulation with pro-inflammatory mediators. Explants were derived from macroscopically normal tendons from 3 horses aged between 9–14 years of age and stimulated with 5 ngml^−1^ IL-1β or combined stimulation with low (0.01 µM) or high (1.0 µM) doses of PGE_2_ with 5 ngml^-1^ IL-1β compared to non-stimulated controls. LXA_4_ release was increased in all stimulated samples compared to respective controls (*P* = 0.005). Treatment with IL-1β induced greater LXA_4_ production compared to controls (*P* = 0.011). Combined stimulation with high dose PGE_2_ enhanced LXA_4_ release compared to low dose PGE_2_ (*P* = 0.032). Error bars represent standard deviation. * *P*<0.05.

Stimulation of tendon explants with either IL-1β or a combination of IL-1β and PGE_2_ enhanced LXA_4_ release in media after 24 hours compared to non-stimulated controls (P = 0.005). Combined stimulation with IL-1β and 1.0 µM PGE_2_ induced higher levels of LXA_4_ release compared to 0.01 µM PGE_2_ and IL-1β (Fig. 8, *P* = 0.32).

## Discussion

Prostaglandins such as PGE_2_ are produced by tenocytes and other fibroblasts in response to injury and after stimulation with pro-inflammatory cytokines [21], [22], initiating MMP mediated catabolism of tendon ECM [40]. Although seemingly destructive to the local tissue architecture, this process facilitates clearance of cellular debris and debridement of the affected ECM as described for wound healing in other connective tissues. Prostaglandins may also exert beneficial regulatory actions in healthy tissues maintaining normal physiologic processes such as local bone remodelling [41] and modification of renal blood flow [42]. Furthermore, their presence following injury signals the onset of lipoxin mediated resolution processes, such that the duration and magnitude of the inflammatory response can be regulated to restrict the degree of tissue damage [33]. Thus prostaglandins can be said to possess ‘double-edged sword’ properties in terms of their dichotomous roles in wound healing processes. The extent to which these properties play a role in tendinopathies with injury and repair stage remains unclear.

In the current study, PGF_2α_ levels were unchanged with injury and were substantially lower than PGE_2_ levels in normal tendons. This may imply differential regulation of these prostaglandins in tendon, with PGF_2α_ less susceptible to changes with injury suggesting PGE_2_ is the main prostaglandin operative in tendon injury. PGE_2_ levels were found to decrease with aging in normal tendons. This could be a consequence of the reduction in tendon cellularity with increasing age [43], [44] leading to a decreasing tendon prostaglandin synthetic capacity. Alternatively, it may be related to a lack of PGE_2_ synthesising pro-inflammatory macrophages as we have described previously for uninjured tendons [16]. The relationship between age and the pattern of PGE_2_ levels was difficult to determine in injured tendons because of the confounding issue that sub-acute injury predominated in younger horses compared to chronic injury, which occurred with greater frequency in older individuals. However, the positive correlation between increasing tendon PGE_2_ levels with age in injured horses could be attributable to a greater PGE_2_ synthetic capacity both by increased tendon fibroblast cellularity and infiltration of pro-inflammatory macrophages into injured regions of tendon [16]. This was supported by the increase in mPGES-1:PGDH ratio in sub-acute injury which suggests an interplay between PGE_2_ synthesis and degradation could lead to an increased synthetic capacity in the tissue. Furthermore, activated macrophages from aged humans and mice are reported to produce more PGE_2_ than macrophages from younger individuals [45] which may contribute to the greater frequency of tendon injury in older individuals through sustained activation of proteolytic action on the ECM. Whilst there are no equine specific antibodies available to neutrophils or mast cells, precluding immunofluorescent analysis, we were not able to identify these cells by histology of injured tendons between 3–6 weeks post injury (data not shown). As we were unable to access tendons with injuries of less than 2 weeks duration, we cannot exclude the presence of these cells and their contribution to the synthesis of PGE_2_ at this earlier phase of injury. However as macrophages are known to release PGE_2_ and tendon injury has been shown to be associated with activation and recruitment of these cells [16], they represent an important source of PGE_2_ during tendon injury.

Regulation of prostaglandin metabolism is not well documented for normal and pathologic tendons, although the majority of circulating prostaglandins are degraded in the pulmonary vasculature via PGDH [32]. However, tissue levels of PGE_2_ are fine-tuned by locally produced PGDH [46] and the net balance between synthesis and degradation may be a mechanism for controlling the action of PGE_2_. In the present study, the ratio of mPGES-1: PGDH was increased in sub-acute compared to chronic disease or normal tendons, suggesting potential aberration of these genes with disease phase. We propose that the altered intracellular prostaglandin regulation is attributable to a proportionately greater increase in mPGES-1 transcription rather than reduced PGDH transcription due to our observation of an increase in PGDH protein in sub-acute tendon injuries. In further support of this, PGDH kinetics have shown it to be a short lived enzyme whose replacement is dependent upon *de novo* protein synthesis at the level of translation rather than that of transcription, due to the prolonged half life of PGDH mRNA [47]. Thus, PGDH mRNA is present in low abundance as a stable moiety, presumably as a mechanism for rapid and precise control of enzyme activity. Although these data suggest PGE_2_ levels should be increased due to elevated mPGES-1 mRNA, the observed reduction in PGE_2_ levels in sub-acute injury compared to normal and chronic injuries could be explained by the increased PGDH protein levels in sub-acute injury. This suggests a secondary (cellular) clearance mechanism for PGE_2_ degradation whereby local PGE_2_ levels can be regulated [46]. The increased vascularity of tendon that occurs after recent injury may also be a contributing factor to the paradoxical lower levels of PGE_2_ after injury [48], as the increased vascular perfusion is likely to facilitate efficient systemic prostaglandin clearance. In addition to vascular clearance, the lower levels of PGE_2_ in sub-acute tendon injury could also be attributable to lipid substrate re-routing towards the resolving pathways that are activated during inflammation. These recently discovered pathways demonstrate critical roles in the switching of lipid mediators from the prostaglandin to the lipoxin axis, returning injured tissues to their previous state [49] by depleting PGE_2_ levels due to reduced arachadonic acid substrate availability. Indeed, the current study shows significantly increased LXA_4_ levels in sub-acute injury compared to normal and chronic injured tendons, suggesting pro-resolving processes are active during the early stage of tendon injury. The alterations in the profile of lipid mediators during this time include low PGE_2_ and elevated LXA_4_ levels compared to normal and chronic injuries and suggest lipid mediator class switching is active in the early phase of tendon injury. We propose this class switching represents an endogenous protective mechanism to limit the degree of damage to tendon ECM and preserve tissue integrity. This concept is supported in part by the findings from this study, demonstrating combined stimulation of normal tendon explants with 5 ngml^-1^ IL-1β and 0.01 µM or 1.0 µM PGE_2_ induced LXA_4_ release, with greater production with the higher dose of PGE_2_. It has been previously shown in an identical experimental system that addition of 1.0 µM PGE_2_ to normal tendon explants induced maximal LXA_4_ release after 72 hours in tissue culture [16]. These observations suggest that PGE_2_ may exert anti-catabolic effects on tendon ECM via the induction of pro-resolving LXA_4_ and switching of lipid mediators from the prostaglandin to the lipoxin axis. Furthermore, in the setting of a pro-inflammatory environment, the presence of higher levels of PGE_2_ may exert an auto-regulatory feedback effect on IL-1 activity in order to modulate the inflammatory reaction [50]. Although the cell types responsible for lipid mediator class switching have not been identified in inflamed tendons, we hypothesise that the interaction between resident tendon cells and infiltrating pro-inflammatory macrophages during early stage injury initiates activation of pro-resolving processes. LXA_4_ levels were reduced during the chronic injury phase where the tendon does not return to normal structure and function. As LXA_4_ is a key determinant of pro-resolving processes [51] it is therefore plausible that incomplete resolution sustains a low level of inflammation, perpetuating chronic disease. Although the present study did not measure the multiple enzymes that synthesise the components of prostaglandin and lipoxin pathways, it is hypothesised that control of class switching involves the regulation of some of these enzymes.

The lipoxin A_4_ receptor FPR2/ALX is reported to have a pivotal role in controlling the duration and magnitude of the inflammatory response, providing endogenous stop signals for inflammation [33], [34]. Despite the anticipated importance of specialised pro-resolving mediators such as LXA_4_ in healing, these resolving pathways are not widely studied in injured tendons. We recently identified significantly increased expression of FPR2/ALX by tenocytes in early equine tendon injury [16] and studies in other inflamed connective tissues have emphasised the importance of resolution processes for regulating inflammation, including inhibition of leukocyte recruitment and modification of vascular permeability [33]. The current study provides novel data illustrating levels of FPR2/ALX are markedly diminished in the tendons of aged injured individuals. Because these mediators are essential for controlling the inflammatory cascade, this suggests an age-related deterioration of tendons to mount a counter-response to inflammation via FPR2/ALX. A component of immunosenescence is ‘inflamm-aging’ whereby aged individuals exhibit diminished ability to modulate inflammation [37], [52]. Studies in humans and rodents report an age related decline in cutaneous wound repair [45], [53], suggesting age contributes to dysregulated tissue repair. Our data of reduced FPR2/ALX together with the elevated PGE_2_ levels with age suggests an inflamm-aging process is present in injured tendons. In support of this concept, IL-1β stimulated tendon explants derived from uninjured horses aged 10 years and above showed a diminished capacity to express FPR2/ALX compared to individuals less than 10 years of age. Interestingly, tendon derived cells from older horses also had a reduced response to IL-1β induced PGE_2_ production compared to young horses (Fig. S1). Taken together these data suggest aged individuals are less capable of mounting a protective response to tendon inflammation by this mechanism compared to younger individuals. Immunosenescence may therefore represent a potential mechanism for tendon re-injury via this pathway and in the development of chronic injury and explain, at least in part, the high frequency of tendon injuries sustained by aged individuals [17], [18]. We hypothesise that ‘inflamm-aging’ is an important component in the complex aetiology of tendinopathy, to which additional contributing factors such as load-induced proteolytic matrix disruption [9] and lower tendon metabolic activity with age [54] are superimposed. Furthermore, as we did not observe any relationship between LXA_4_ levels with age in samples of natural disease or *in vitro*, it appears that it is the ability to generate the FPR2/ALX receptor that is impeded by age and not synthesis of the LXA_4_ ligand, diminishing the ability to mediate downstream processes culminating in a deficit to resolve tendon inflammation.

From the findings in the present study, we conclude that PGE_2_ is implicated in the development of tendon inflammation and its ensuing resolution. Temporal analysis suggests class switching of lipid mediators occurs in early stage injury, driving synthesis of LXA_4_. However, ageing individuals exhibit a reduced capacity to resolve tendon inflammation via the FPR2/ALX receptor, which may present a mechanism for the development of chronic tendinopathy. Improved understanding of injury pathogenesis throughout the phases of tendon repair will facilitate identification of novel therapeutic targets to modulate or prevent disease progression, such as the use of FPR2/ALX agonists.

## Materials and Methods

### Ethics Statement

Ethical approval for the collection of post mortem equine tendons from an abattoir or local equine veterinary referral hospital for this study was sought and approved from the Ethics and Welfare Committee at the Royal Veterinary College (URN 2011 1117).

### Classification of Injury Stage

Equine forelimbs from Thoroughbred or Thoroughbred cross breed horses aged between 2–20 years were obtained from an abattoir or local equine referral hospital with known history of injury and the tensile region of the SDFT harvested within 4 hours of death. Tendons were grouped as sub-acutely injured (3–6 weeks post injury, n = 6, mean age 9±5 years) or chronically injured (>3 months post injury, n = 9, mean age 13±4 years), as shown before [16]. Tendon injuries were aged based on historical information obtained from either the owner or referring veterinary surgeon prior to euthanasia of the horse. Tendons were classified as normal based on their macroscopic post mortem appearance which included lack of visible signs of swelling of the tendon body and a consistent pattern of fascicles on transverse sections (n = 19, mean age 8±5 years). The typical microscopic appearance of normal, sub-acute and chronic injured tendons are shown in Fig. 1.

### Determination of Prostaglandin Levels in Tendons

After harvest, samples were stored at −80°C and assayed within 6 months. Tendon extracts were prepared as described by Zhang and Wang [55]. Briefly, approximately 1 g of the central portion of the tensile region of the SDFT was cut into 5 mm^3^ cubes. Tissue was placed in 5 ml of homogenization buffer (0.1 M Phosphate Buffered Saline (PBS) (PAA, UK) pH 7.4, containing 1 mM EDTA and 10 µM Indomethacin) and homogenized using a dismembranator (Retsch MM2000, Germany). Acetone (2 ml) was added and the sample vortexed and allowed to stand at room temperature for 5 minutes. Tissue was pelleted by centrifugation at 16,000×g and the supernatant harvested. Acetone was removed by evaporation at room temperature for 18 hours and the residual supernatant used for determination of PGE_2_ and PGF_2α_ measurement via radioimmunoassay as described by Cheng *et al*. [56].

### Measurement of Lipoxin A_4_ in Tendon Extracts and Tissue Culture Media

LXA_4_ levels were determined as an indicator of resolution of inflammation in the supernatant prepared earlier to determine prostaglandin levels in extracts of normal (n = 8), sub-acute (n = 7) and chronic injured SDFTs (n = 6) and in samples of tissue culture media from cytokine stimulated tendon explants as described later. LXA_4_ was separated from the supernatant used to determine prostaglandin levels by passage through Bond Elut C18 columns (Agilent Technologies, USA) followed by elution with methyl formate. Samples were evaporated to dryness in a stream of nitrogen and the resulting residues used to determine LXA_4_ levels by ELISA (Neogen Corp, USA) according to the manufacturer’s instructions. LXA_4_ levels in culture media samples (see section on tendon explant culture below) were determined in an identical manner. The ELISA kit is specific for LXA_4_ showing minimal cross-reactivity [LXA_4_ 100%, Lipoxin B_4_ 1.0%, 15-hydroxyeicosatetraenoic acid (HETE) 0.1%, 5-HETE <0.1% and 12 HETE <0.1% as determined by the supplier].

### Quantitative RT-PCR Analysis of PGE_2_ Synthesis and Degradation Enzymes

RNA was extracted from 200 mg of tendon tissue (normal n = 6, sub-acute, n = 8 and chronic injury n = 6) as described by Young *et al.* using the RNeasy kit (Qiagen, UK) [44]. RNA (22 µL) was used for cDNA synthesis using random primers (Promega, UK) and Superscript II reverse transcriptase (Invitrogen, UK) as described by Young *et al.* Gene specific primers (Table 1) were used to prepare amplicons which were cloned into a pGEM®-T Easy Vector (Promega) and plasmid DNA was used to prepare standard curves as described previously for subsequent absolute copy number evaluation [57]. We investigated expression levels of COX-2, mPGES-1 (inducible terminal enzyme in PGE_2_ synthesis), PGDH and the PGE_2_ receptor EP_4_ which is reported to be implicated in the pathogenesis of tendinopathy [31]. Expression levels of GAPDH and 18S ribosomal RNA were used for normalization. Equine oligonucleotide sequences used for quantitative real-time PCR are shown in Table 1. For each gene of interest and housekeeping gene, 1 µg of cDNA and 1 µM of each forward and reverse primer were used per reaction (conducted in triplicate) and amplified using SYBR® Green JumpStart™ *Taq* ReadyMix™ (Sigma-Aldrich, UK) for quantitative PCR using an Opticon II DNA engine thermocycler (MJ Research, UK). Standard curves ranged from 1×10^8^ to 1×10^1^ copies (GAPDH) or 1×10^10^ to 1×10^3^ copies (18 S) such that absolute copy number could be calculated according to cycle threshold. PCR efficiency was tested in each experiment and confirmed to be approximately 2.0, indicating 100% amplification efficiency according to a previously described mathematical model [58].

**Table 1:** Equine oligonucleotide sequences used for quantitative real-time PCR.

Primer		5′ to 3′ sequence	Annealing temperature (°C)	Amplicon size (bp)
**COX-2**	FwdRev	GAT CCT AAG CGA GGT CCA GC TTC CGT CCT TGA AAA GGC GC	58	116
**EP4r**	FwdRev	TGG TCA TCT TAC TCA TCG CCA CCT TTC ACA GAA GCA ATT CGG ATG GCC	68	154
**mPGES-1**	FwdRev	CAC CGG AAC GAC ATG GAG AC TTC AGC TTG CCC AGG TAG GC	56	152
**PGDH**	FwdRev	GGT GTC TGT TAT CAG TGG AAC C AGG CAC AAT AAA CAG GCT GC	56	137
**GAPDH**	FwdRev	CAG AAC ATC ATC CCT GCT TCT A AGA TCC ACG ACT GAC ACG TTA G	60	125
**18S**	FwdRev	TAG AGG GAC AAG TGG CGT TC CCG AAG AAG ACC ATG CAG CC	55	103

### Western Blot Analysis for mPGES-1 and PGDH

To assess if mRNA changes in PGE_2_ metabolism were reflected at the protein level, Western blotting was performed to assess mPGES-1 and PGDH protein expression in tendon extracts. Protein extracts of normal (n = 7), sub-acute (n = 5) and chronic injured tendons (n = 8) were prepared by extraction of 100 mg of finely diced tendon in 15 volumes of 4 M Guanidine hydrochloride with protease inhibitor cocktail III (Merck, Calbiochem®, UK) for 48 hours at room temperature. Insoluble material was separated by centrifugation (13,000×g, 20 mins) and proteins precipitated by the addition of 9 volumes (v/v) ethanol buffer (ethanol with 50 mM sodium acetate) and chilling to −80°C for 2 hours. Pellets were washed twice with ethanol buffer and dried pellets resuspended in Laemmli sample buffer (Bio-Rad, UK). Samples were reduced by addition of DTT to 0.1 M and heated to 95°C prior to electrophoresis on 10% SDS-polyacrylamide gels. Proteins were transferred onto PVDF membrane (GE Healthcare) and blocked for 2 hours in Tris buffered saline in 1% Triton (TBST buffer) containing 8% powdered skimmed milk and 2% bovine serum albumin (BSA). Membranes were incubated with either anti-human PGDH (Santa Cruz Biotechnology Inc, USA) (diluted 1∶200), anti-human mPGES-1 (Agrisera, Sweden) (diluted 1∶500) or anti-mouse β actin (Sigma-Aldrich) (diluted 1∶2000) antibodies for 2 hours at room temperature in TBST containing 5% BSA and 1% Tween-20®. Washed membranes were incubated with anti-rabbit (diluted 1∶1000, Cell Signaling Technology®, USA) or anti-mouse (diluted 1∶2000, GE Healthcare, UK) secondary antibodies conjugated to horseradish peroxidase for 2 hours to visualise proteins using ECL reagent and film (GE Healthcare, UK) according to manufacturer’s instructions. Densitometry analysis of bands was performed using ImageJ software (NIH) for the protein of interest relative to β-actin.

### Analysis of FPR2/ALX Expression by Immunohistochemistry

Having assessed levels of secreted lipid mediators produced at different stages of tendon injury, we next investigated the ability of injured tissues to mount a resolution response to inflammation. This was accomplished by analyzing FPR2/ALX expression in samples of natural tendon injury and by IL-1β stimulation of normal tendon explants *in vitro*, such that the effect of injury stage and age on the ability to resolve tendon inflammation could be determined. Fresh tendon pieces were embedded in optimal cutting temperature compound (OCT, Sakura Tissue-Tek®, The Netherlands) and snap frozen in pre-chilled (−80°C) n-hexane and stored at −80°C until used. Serial sections (8–10 µm thickness) were cut on a cryostat (Bright, UK), mounted onto poly-L-lysine coated slides (VWR, UK) and allowed to dry for 2 hours at room temperature prior to immunofluorescent analysis as described below. To investigate the influence of horse age on the ability of tendon cells to resolve inflammation via this mechanism, we assessed the effect of IL-1β on FPR2/ALX expression in an explant culture model. Explant tissues from normal tendons were grouped according to horse age as <10 (n = 5) or ≥10 years of age (n = 8). 0.4 cm^3^ explants (300 mg ±30 mg) were incubated at 37°C and 5% CO_2_ under humidified atmosphere in 3 ml of Dulbecco modified Eagle’s medium (PAA, UK) containing 1% Penicillin and Streptomycin without foetal calf serum. Samples were stimulated with 5 ngml^-1^ human recombinant IL-1β (Merck, Calbiochem®, UK) and non-stimulated (vehicle only) samples served as controls. At 72 hours after stimulation, explant tissues were embedded in OCT and snap frozen in chilled (−80°C) n-hexane, and cryosections cut as described above.

Subsequently, consecutive cryosections were blocked in 5% normal goat serum (Sigma-Aldrich) in PBS for 1 hour in a humid chamber and probed with a 1∶100 diluted mouse monoclonal antibody to FPR2/ALX (Lipoxin A_4_ receptor, IgG_1_, AbCam, UK) and secondary goat anti-mouse IgG_1_ (Southern Biotech, USA) each for 2 hours at room temperature. To visualise nuclei, slides were incubated with 0.5 µgml^−1^ Hoechst 33342 (Invitrogen, UK) for 20 minutes and washed in PBS Tween-20® (PBS-T). To quench ***the background fluorescence, slides were incubated*** with 0.1% Sudan Black B (BDH, Poole, UK) in 70% ethanol for 20 minutes [59], washed in PBS-T and mounted under coverslips using a solution of 80% glycerol in 0.5 mM Tris buffer (pH 7.2). Slides were stored at 4°C in the dark until image acquisition. Cryosections of equine spleen were used as positive control tissue to validate suitability and dilution of antibodies for use on tendon sections. Negative controls consisted of spleen cryosections incubated with murine isotype matched primary control antibodies (Southern Biotech, USA) as previously reported [16]. To assess FPR2/ALX expression, images were recorded using a Leica SP5 confocal microscope (Leica Microsystems, UK) as reported elsewhere [16].

### Measurement of LXA_4_ in Media from Explants Stimulated with IL-1*β* and PGE_2_

Our previous work has shown that stimulation of normal tendon explants with 5 ngml^−1^ IL-1β or 1.0 µM PGE_2_ *in vitro* induced production of the pro-resolving ligand LXA_4_ which binds to FPR2/ALX [16]. In the current study, LXA_4_ levels in media were measured to ascertain if concurrent stimulation of macroscopically normal tendon explants with IL-1β (5 ngml^−1^) and low (0.01 µM) or high dose (1.0 µM) PGE_2_ induced a dose-dependent increase in LXA_4_ levels. Tendon explants were derived from 3 horses aged between 9 and 14 years and LXA_4_ levels determined 24 hours after stimulation with the combination of pro-inflammatory mediators.

### Statistical Analysis

Statistical analyses were performed using GraphPad Prism 5 (GraphPad Software Inc., San Diego, CA). Normality was tested using a Kolmogorov-Smirnov test. One-way ANOVA with Tukey’s multiple comparison tests were performed to determine differences in PGE_2_, LXA_4_ and the ratio of PGDH to β-actin protein between normal, sub-acute and chronic injured tendons. Kruskal-Wallis tests were performed to compare gene expression of mPGES-1, PGDH, COX-2 and the EP_4_ receptor normalized to housekeeping genes in normal, sub-acute and chronic injured tendons. Kruskal-Wallis with post hoc Mann Whitney tests were used to compare gene ratios of mPGES-1 to PGDH in normal, sub-acute and chronic injured tendons. A Mann Whitney test was used to detect differences in FPR2/ALX expression in IL-1β stimulated tendon explants *in vitro* from horses <10 or ≥ 10 years of age. Relationships between horse age and PGE_2_ levels or FPR2/ALX expression in normal and injured tendons were assessed by linear correlation analysis. A linear mixed model using SPSS PASW Statistics 18 (SPSS Inc Illinois, USA) was used to analyse LXA_4_ release from tendon explants stimulated with pro-inflammatory mediators to account for effects of horse and experimental condition. In all cases, the *P* value was considered significant if below 0.05.

## Supporting Information

Figure S1 **Prostaglandin E_2_ (PGE_2_) production by tendon derived cells stimulated with IL-1β (5 ngml^-1^)** ***in vitro*** **.** Tendon cells derived from 8 year old horses (n = 3) had a reduced response to IL-1β induced PGE_2_ production compared to 3 year old horses (n = 3). Median values are shown with maximum and minimum range.
(TIF)

## References

[1] Kujala UM, Sarna S, Kaprio J (2005) Cumulative incidence of achilles tendon rupture and tendinopathy in male former elite athletes. Clin J Sport Med 15: 133–135. 15867554 10.1097/01.jsm.0000165347.55638.23

[2] Avella CS, Ely ER, Verheyen KL, Price JS, Wood JL, et al (2009) Ultrasonographic assessment of the superficial digital flexor tendons of National Hunt racehorses in training over two racing seasons. Equine Vet J 41: 449–454. 19642404 10.2746/042516409x391042

[3] Almekinders LC, Temple JD (1998) Etiology, diagnosis, and treatment of tendonitis: an analysis of the literature. Med Sci Sports Exerc 30: 1183–1190. 9710855 10.1097/00005768-199808000-00001

[4] Birch HL, Bailey AJ, Goodship AE (1998) Macroscopic ‘degeneration’ of equine superficial digital flexor tendon is accompanied by a change in extracellular matrix composition. Equine Vet J 30: 534–539. 9844973 10.1111/j.2042-3306.1998.tb04530.x

[5] Dyson SJ (2004) Medical management of superficial digital flexor tendonitis: a comparative study in 219 horses (1992–2000). Equine Vet J 36: 415–419. 15253082 10.2746/0425164044868422

[6] Crevier-Denoix N, Collobert C, Pourcelot P, Denoix JM, Sanaa M, et al.. (1997) Mechanical properties of pathological equine superficial digital flexor tendons. Equine Vet J Suppl: 23–26. 10.1111/j.2042-3306.1997.tb05046.x 9354282

[7] Alfredson H, Lorentzon R (2002) Chronic tendon pain: no signs of chemical inflammation but high concentrations of the neurotransmitter glutamate. Implications for treatment? Curr Drug Targets 3: 43–54. 11899264 10.2174/1389450023348028

[8] Kannus P, Jozsa L (1991) Histopathological changes preceding spontaneous rupture of a tendon. A controlled study of 891 patients. J Bone Joint Surg Am 73: 1507–1525. 1748700

[9] Dudhia J, Scott CM, Draper ER, Heinegard D, Pitsillides AA, et al (2007) Aging enhances a mechanically-induced reduction in tendon strength by an active process involving matrix metalloproteinase activity. Aging Cell 6: 547–556. 17578513 10.1111/j.1474-9726.2007.00307.x

[10] Strocchi R, De Pasquale V, Guizzardi S, Govoni P, Facchini A, et al (1991) Human Achilles tendon: morphological and morphometric variations as a function of age. Foot Ankle 12: 100–104. 1773989 10.1177/107110079101200207

[11] Wilson AM, McGuigan MP, Su A, van Den Bogert AJ (2001) Horses damp the spring in their step. Nature 414: 895–899. 11780059 10.1038/414895a

[12] Ker RF, Wang XT, Pike AV (2000) Fatigue quality of mammalian tendons. J Exp Biol 203: 1317–1327. 10729280 10.1242/jeb.203.8.1317

[13] Dowling BA, Dart AJ, Hodgson DR, Smith RK (2000) Superficial digital flexor tendonitis in the horse. Equine Vet J 32: 369–378. 11037257 10.2746/042516400777591138

[14] Stephens PR, Nunamaker DM, Butterweck DM (1989) Application of a Hall-effect transducer for measurement of tendon strains in horses. Am J Vet Res 50: 1089–1095. 2774333

[15] Riemersma DJ, Schamhardt HC (1985) In vitro mechanical properties of equine tendons in relation to cross-sectional area and collagen content. Res Vet Sci 39: 263–270. 4081329

[16] Dakin SG, Werling D, Hibbert A, Abayasekara DR, Young NJ, et al (2012) Macrophage sub-populations and the lipoxin A4 receptor implicate active inflammation during equine tendon repair. PLoS One 7: e32333. 22384219 10.1371/journal.pone.0032333 PMC3284560

[17] Kasashima Y, Takahashi T, Smith RK, Goodship AE, Kuwano A, et al (2004) Prevalence of superficial digital flexor tendonitis and suspensory desmitis in Japanese Thoroughbred flat racehorses in 1999. Equine Vet J 36: 346–350. 15163043 10.2746/0425164044890580

[18] Moller A, Astron M, Westlin N (1996) Increasing incidence of Achilles tendon rupture. Acta Orthop Scand 67: 479–481. 8948254 10.3109/17453679608996672

[19] Maffulli N, Waterston SW, Squair J, Reaper J, Douglas AS (1999) Changing incidence of Achilles tendon rupture in Scotland: a 15-year study. Clin J Sport Med 9: 157–160. 10512344 10.1097/00042752-199907000-00007

[20] Tsuzaki M, Guyton G, Garrett W, Archambault JM, Herzog W, et al (2003) IL-1 beta induces COX2, MMP-1, -3 and -13, ADAMTS-4, IL-1 beta and IL-6 in human tendon cells. J Orthop Res 21: 256–264. 12568957 10.1016/S0736-0266(02)00141-9

[21] Khan MH, Li Z, Wang JH (2005) Repeated exposure of tendon to prostaglandin-E2 leads to localized tendon degeneration. Clin J Sport Med 15: 27–33. 15654188 10.1097/00042752-200501000-00006

[22] Yang G, Im HJ, Wang JH (2005) Repetitive mechanical stretching modulates IL-1beta induced COX-2, MMP-1 expression, and PGE2 production in human patellar tendon fibroblasts. Gene 363: 166–172. 16226404 10.1016/j.gene.2005.08.006 PMC2901527

[23] Tilley SL, Coffman TM, Koller BH (2001) Mixed messages: modulation of inflammation and immune responses by prostaglandins and thromboxanes. J Clin Invest 108: 15–23. 11435451 10.1172/JCI13416 PMC209346

[24] Langberg H, Skovgaard D, Karamouzis M, Bulow J, Kjaer M (1999) Metabolism and inflammatory mediators in the peritendinous space measured by microdialysis during intermittent isometric exercise in humans. J Physiol 515 (Pt 3): 919–927. 10.1111/j.1469-7793.1999.919ab.x PMC2269174 10066916

[25] Zhang J, Wang JH (2010) Production of PGE(2) increases in tendons subjected to repetitive mechanical loading and induces differentiation of tendon stem cells into non-tenocytes. J Orthop Res 28: 198–203. 19688869 10.1002/jor.20962

[26] Almekinders LC, Banes AJ, Ballenger CA (1993) Effects of repetitive motion on human fibroblasts. Med Sci Sports Exerc 25: 603–607. 8388071

[27] Almekinders LC, Baynes AJ, Bracey LW (1995) An in vitro investigation into the effects of repetitive motion and nonsteroidal antiinflammatory medication on human tendon fibroblasts. Am J Sports Med 23: 119–123. 7726341 10.1177/036354659502300120

[28] Wang JH, Li Z, Yang G, Khan M (2004) Repetitively stretched tendon fibroblasts produce inflammatory mediators. Clin Orthop Relat Res: 243–250. 10.1097/01.blo.0000126337.65685.e4 15187863

[29] Ruwanpura SM, Noguchi K, Ishikawa I (2004) Prostaglandin E2 regulates interleukin-1beta-induced matrix metalloproteinase-3 production in human gingival fibroblasts. J Dent Res 83: 260–265. 14981131 10.1177/154405910408300315

[30] Attur M, Al-Mussawir HE, Patel J, Kitay A, Dave M, et al (2008) Prostaglandin E2 exerts catabolic effects in osteoarthritis cartilage: evidence for signaling via the EP4 receptor. J Immunol 181: 5082–5088. 18802112 10.4049/jimmunol.181.7.5082

[31] Thampatty BP, Li H, Im HJ, Wang JH (2007) EP4 receptor regulates collagen type-I, MMP-1, and MMP-3 gene expression in human tendon fibroblasts in response to IL-1 beta treatment. Gene 386: 154–161. 17046175 10.1016/j.gene.2006.08.027 PMC1839868

[32] Hahn EL, Gamelli RL (2000) Prostaglandin E2 synthesis and metabolism in burn injury and trauma. J Trauma 49: 1147–1154. 11130507 10.1097/00005373-200012000-00033

[33] Serhan CN, Takano T, Chiang N, Gronert K, Clish CB (2000) Formation of endogenous “antiinflammatory” lipid mediators by transcellular biosynthesis. Lipoxins and aspirin-triggered lipoxins inhibit neutrophil recruitment and vascular permeability. Am J Respir Crit Care Med 161: S95–S101. 10673235 10.1164/ajrccm.161.supplement_1.ltta-19

[34] Chiang N, Serhan CN, Dahlen SE, Drazen JM, Hay DW, et al (2006) The lipoxin receptor ALX: potent ligand-specific and stereoselective actions in vivo. Pharmacol Rev 58: 463–487. 16968948 10.1124/pr.58.3.4

[35] Serhan CN, Hamberg M, Samuelsson B (1984) Lipoxins: novel series of biologically active compounds formed from arachidonic acid in human leukocytes. Proc Natl Acad Sci U S A 81: 5335–5339. 6089195 10.1073/pnas.81.17.5335 PMC391698

[36] Takano T, Fiore S, Maddox JF, Brady HR, Petasis NA, et al (1997) Aspirin-triggered 15-epi-lipoxin A4 (LXA4) and LXA4 stable analogues are potent inhibitors of acute inflammation: evidence for anti-inflammatory receptors. J Exp Med 185: 1693–1704. 9151906 10.1084/jem.185.9.1693 PMC2196289

[37] Franceschi C, Bonafe M, Valensin S, Olivieri F, De Luca M, et al (2000) Inflamm-aging. An evolutionary perspective on immunosenescence. Ann N Y Acad Sci 908: 244–254. 10911963 10.1111/j.1749-6632.2000.tb06651.x

[38] Jarvinen TA, Kannus P, Maffulli N, Khan KM (2005) Achilles tendon disorders: etiology and epidemiology. Foot Ankle Clin 10: 255–266. 15922917 10.1016/j.fcl.2005.01.013

[39] Sayasith K, Bouchard N, Dore M, Sirois J (2007) Cloning of equine prostaglandin dehydrogenase and its gonadotropin-dependent regulation in theca and mural granulosa cells of equine preovulatory follicles during the ovulatory process. Reproduction 133: 455–466. 17307913 10.1530/REP-06-0210

[40] Jones GC, Corps AN, Pennington CJ, Clark IM, Edwards DR, et al (2006) Expression profiling of metalloproteinases and tissue inhibitors of metalloproteinases in normal and degenerate human achilles tendon. Arthritis Rheum 54: 832–842. 16508964 10.1002/art.21672

[41] Graham S, Gamie Z, Polyzois I, Narvani AA, Tzafetta K, et al (2009) Prostaglandin EP2 and EP4 receptor agonists in bone formation and bone healing: In vivo and in vitro evidence. Expert Opin Investig Drugs 18: 746–766. 10.1517/13543780902893051 19426119

[42] Dunn M (1987) The role of arachidonic acid metabolites in renal homeostasis. Non-steroidal anti-inflammatory drugs renal function and biochemical, histological and clinical effects and drug interactions. Drugs 33 Suppl 156–66. 3109872 10.2165/00003495-198700331-00009

[43] Stanley RL, Fleck RA, Becker DL, Goodship AE, Ralphs JR, et al (2007) Gap junction protein expression and cellularity: comparison of immature and adult equine digital tendons. J Anat 211: 325–334. 17848160 10.1111/j.1469-7580.2007.00781.x PMC2375813

[44] Young NJ, Becker DL, Fleck RA, Goodship AE, Patterson-Kane JC (2009) Maturational alterations in gap junction expression and associated collagen synthesis in response to tendon function. Matrix Biol 28: 311–323. 19481603 10.1016/j.matbio.2009.05.002

[45] Plowden J, Renshaw-Hoelscher M, Engleman C, Katz J, Sambhara S (2004) Innate immunity in aging: impact on macrophage function. Aging Cell 3: 161–167. 15268749 10.1111/j.1474-9728.2004.00102.x

[46] Fincham N, Camp R (1983) Novel prostaglandin dehydrogenase in rat skin. Biochem J 212: 129–134. 6575778 10.1042/bj2120129 PMC1152019

[47] Blackwell GJ, Flower RJ, Vane JR (1975) Rapid reduction of prostaglandin 15-hydroxy dehydrogenase activity in rat tissues after treatment with protein synthesis inhibitors. Br J Pharmacol 55: 233–238. 1201382 10.1111/j.1476-5381.1975.tb07633.x PMC1666836

[48] Stromberg B (1973) Morphologic, thermographic and 133Xe clearance studies on normal and diseased superficial digital flexor tendons in race horses. Equine Vet J 5: 156–161. 4766689 10.1111/j.2042-3306.1973.tb03217.x

[49] Levy BD, Clish CB, Schmidt B, Gronert K, Serhan CN (2001) Lipid mediator class switching during acute inflammation: signals in resolution. Nat Immunol 2: 612–619. 11429545 10.1038/89759

[50] Knudsen PJ, Dinarello CA, Strom TB (1986) Prostaglandins posttranscriptionally inhibit monocyte expression of interleukin 1 activity by increasing intracellular cyclic adenosine monophosphate. J Immunol 137: 3189–3194. 3021848

[51] Serhan CN, Chiang N (2002) Lipid-derived mediators in endogenous anti-inflammation and resolution: lipoxins and aspirin-triggered 15-epi-lipoxins. ScientificWorldJournal 2: 169–204. 10.1100/tsw.2002.81 PMC6009744 12806051

[52] Desai A, Grolleau-Julius A, Yung R Leukocyte function in the aging immune system. J Leukoc Biol 87: 1001–1009. 10.1189/jlb.0809542 PMC4057658 20200405

[53] Gosain A, DiPietro LA (2004) Aging and wound healing. World J Surg 28: 321–326. 14961191 10.1007/s00268-003-7397-6

[54] Almekinders LC, Deol G (1999) The effects of aging, antiinflammatory drugs, and ultrasound on the in vitro response of tendon tissue. Am J Sports Med 27: 417–421. 10424209 10.1177/03635465990270040301

[55] Zhang J, Wang JH (2010) Production of PGE(2) increases in tendons subjected to repetitive mechanical loading and induces differentiation of tendon stem cells into non-tenocytes. J Orthop Res 28: 198–203. 19688869 10.1002/jor.20962

[56] Cheng Z, Robinson RS, Pushpakumara PG, Mansbridge RJ, Wathes DC (2001) Effect of dietary polyunsaturated fatty acids on uterine prostaglandin synthesis in the cow. J Endocrinol 171: 463–473. 11739012 10.1677/joe.0.1710463

[57] Young NJ, Thomas CJ, Collins ME, Brownlie J (2006) Real-time RT-PCR detection of Bovine Viral Diarrhoea virus in whole blood using an external RNA reference. J Virol Methods 138: 218–222. 17030066 10.1016/j.jviromet.2006.08.008 PMC7112878

[58] Pfaffl MW (2001) A new mathematical model for relative quantification in real-time RT-PCR. Nucleic Acids Res 29: e45. 11328886 10.1093/nar/29.9.e45 PMC55695

[59] Romijn HJ, van Uum JF, Breedijk I, Emmering J, Radu I, et al (1999) Double immunolabeling of neuropeptides in the human hypothalamus as analyzed by confocal laser scanning fluorescence microscopy. J Histochem Cytochem 47: 229–236. 9889258 10.1177/002215549904700211

