# Supplementary figures and images for: Inflamm-Aging and Arachadonic Acid Metabolite Differences with Stage of Tendon Disease
Source: PLoS One. 2012 Nov 14;7(11):e48978. doi: 10.1371/journal.pone.0048978 (PMC3498370; doi:10.1371/journal.pone.0048978)

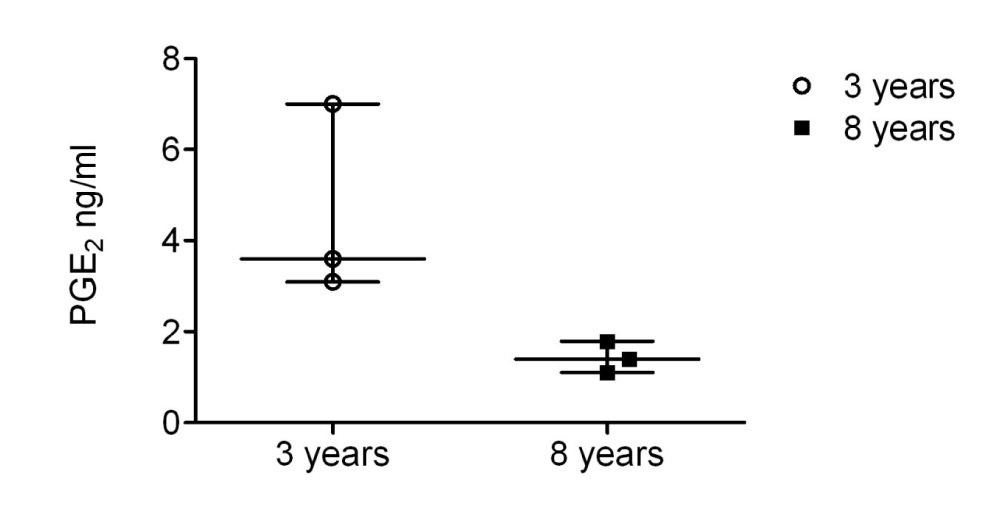

Supplement: Figure S1 — Prostaglandin E2 (PGE2) production by tendon derived cells stimulated with IL-1β (5 ngml-1) in vitro . Tendon cells derived from 8 year old horses (n = 3) had a reduced response to IL-1β induced PGE2 production compared to 3 year old horses (n = 3). Median values are shown with maximum and minimum range. (TIF) [file pone.0048978.s001.tif]
